# Biodegradation of Phenol by Bacteria Strain *Acinetobacter Calcoaceticus* PA Isolated from Phenolic Wastewater

**DOI:** 10.3390/ijerph13030300

**Published:** 2016-03-08

**Authors:** Zhenghui Liu, Wenyu Xie, Dehao Li, Yang Peng, Zesheng Li, Shusi Liu

**Affiliations:** 1 Department of Environmental Engineering, School of Environmental and Biological Engineering, Guangdong University of Petrochemical Technology, Maoming 525000, China; lzhhok@126.com (Z.L.); dehlee@163.com (D.L.); 15968442034@163.com (Y.P.); 18814090425@163.com (S.L.); 2 Technology Research Center for Petrochemical Resources Clean Utilization of Guangdong Province, Maoming 525000, China; lzs212@163.com

**Keywords:** phenol-degrading, biodegradation, *Acinetobacter calcoaceticus*

## Abstract

A phenol-degrading bacterium strain PA was successfully isolated from the effluent of petrochemical wastewater. Based on its morphological, physiological and biochemical characteristics, the strain PA was characterized as a Gram-negative, strictly aerobic, nonmotile and short rod-shaped bacterium that utilizes phenol as a sole carbon and energy source. 16S rDNA sequence analysis revealed that this strain is affiliated to *Acinetobacter calcoaceticus* in the group of *Gammaproteobacteria*. The strain was efficient in removing 91.6% of the initial 800 mg∙L^−1^ phenol within 48 h, and had a tolerance of phenol concentration as high as 1700 mg∙L^−1^. These results indicated that *A. calcoaceticus* possesses a promising potential in treating phenolic wastewater.

## 1. Introduction

The massive production and application of phenol in industrial activities make it a major environmental pollutant in most wastewater various facilities, such as oil refineries, coking plants, pharmaceuticals and plastic industries [1,2]. Many aquatic organisms, including microorganisms, plants and fishes, pose a risk of mutagenic, teratogenic and carcinogenic effects in the environments that contain phenol pollutants. Thus, phenol has been classified as a highly hazardous chemical [3], and has been included in the list of priority pollutants by the U.S. Environmental Protection Agency [4]. Finding an efficient method to remove phenol effectively has attracted increasing concern for the environmental remediation and the health welfare of human beings.

Physico-chemical methods, such as ultra violet, ozonation, hydrogen peroxide, Fenton’s reagent, or a combination of these methods, have been employed to eliminate phenol in industrial wastewater [5,6]. These methods were effective in removing phenol but were complex and costly. Hence, the development of improved technologies capable of degrading phenol in the environments is necessary. Alternatively, the biodegradation of phenols is an environmentally friendly and cost-effective technology currently preferred to reduce phenol compounds [7]. 

Based on the theory that a few microorganisms can utilize phenol as the sole source of carbon and energy [8,9], numerous bacterial species have been isolated and characterized as phenol-degrading microorganisms [10,11]. These bacterial species include *Pseudomonas putida* [12], *Rhodococcus erythropolis* [13], *Bacillus subtilis* [14], *Bacillus brevis* [11], *Serratia marcescens* [15], *Arthrobacter citreus* [16], *Alcaligenes faecalis* [17], *Sphingomonas* [18], and *Acinetobacter* [19,20,21]. Native microbial species have been reported to be more adaptive and capable of out-competing non-indigenous microorganisms in the remediation of special polluted environments [22]. Therefore, identifying new phenol-degrading bacteria is necessary for the bioremediation of the phenol-contaminated environments in various regions. 

In the current study, a bacterial strain capable of degrading phenol was isolated from the petrochemical wastewater in South China. The strain PA was affiliated to *Acinetobacter calcoaceticus* in the group of *Gammaproteobacteria.* The degradation efficiencies of phenol for the strain were then examined. In addition, the growth limitation of the strain in elevated phenol concentrations was evaluated. 

## 2. Materials and Methods 

### 2.1. Chemicals, Media and Phenolic Wastewater Sample

All chemicals used were analytical reagents. The minimal salt media (MSM) and Luria-Bertani (LB) media were used in present study. The MSM contained KH_2_PO_4_ 0.5 g, K_2_HPO_4_ 0.5 g, CaCl_2_ 0.1 g, NaCl 0.2 g, MgSO_4_·7H_2_O 0.5 g, MnSO_4_·7H_2_O 0.01 g, FeSO_4_·7H_2_O 0.01 g, NH_4_NO_3_ 1.0 g per liter. The LB media was composed of tryptone 10 g, yeast extract 5 g and NaCl 5 g per liter. Deionized, distilled water was used for the experiments. The phenolic wastewaster samples along with activated sludge were collected in a biological treatment system from a petrochemical company in Guangdong, China. The phenol concentration in the wastewater was approximately 100 mg·L^−1^.

### 2.2. Enrichment and Isolation of Phenol-Degrading Bacteria

The phenolic wastewater samples collected from an oil refinery effluent in South China was inoculated into flasks containing MSM for the enrichment culture. Phenol was supplemented in the media as the sole carbon source, and the various concentrations of phenol were 200, 500, 800, 1100, 1400, 1700, 2000 and 2500 mg·L^−1^. The enriched culture observed with more biomass (in the flask with 800 mg·L^−1^ phenol) was further transferred into a freshly prepared enrichment media with higher concentrations of phenol (increased from 800 mg·L^−1^ to 2500 mg·L^−1^). The final enriched media were diluted serially and spread on LB agar plates supplemented with phenol (500 mg·L^−1^). The plates were incubated at 30 °C and single colonies with morphological differences were selected and streaked on new plates. The resulting isolates were stored at 4 °C for further study.

The morphological properties of the isolated colonies were observed by optical microscopy. The typical physiological and biochemical characteristics of the phenol-degrading bacteria strains, such as Gram’s staining, motility, starch hydrolysis, and gelatinase [23] were systematically performed according to Bergey’s manual of determinative of bacteriology [24]. Indole test, methyl red test and hydrogen sulfide test were also analyzed, as described previously [25].

### 2.3. Electron Microscopy

SEM (scanning electron microscopy): Bacterial cell morphologies of phenol-degrading strains were examined by scanning electron microscope S-3000N (Hitachi, Tokyo, Japan). For the preparations, cells were fixed in 0.1 M phosphate buffer solution (PBS) containing 2.5% (v/v) glutaraldehyde at 4 °C for 5 h. The samples were then washed with PBS three times (10 min each time). For dehydration, the samples were treated with ethanol serials (30%, 50%, 70%, 90%, and 100%, v/v) for 20 min each. Dehydrated cells were filtered through a 0.2 μM polycarbonate filter, dried with a CO_2_-critical point dryer, coated with gold and observed subsequently by SEM at 20 kV. 

TEM (transmission electron microscopy): The strain PA cells were harvested by centrifugation, washed with distilled water, and then placed onto carbon-coated nickel grids. After the cells were settled on the grid for 10 min, the excess liquid was drained off with filter paper, and the samples were air-dried. Subsequently, observations were preformed with a transmission electron microscope H-7650 (Hitachi, Tokyo, Japan) operating at 80 kV.

### 2.4. Characterization by 16S rDNA and Phylogenetic Analysis

The genomic DNA of strain PA was extracted as previously described [26]. DNA was used as template to amplify bacterial 16S rDNA with universal primers 27F (5′-AGAGATTGATCCTGGCTCTG-3′) and 1492R (5′-GGTTTCCTTGTTACGACAT-3′) on a Mastercycler gradient thermocycler (Eppendorf, Hamburg, Germany). The amplification reaction was performed in 25 μL volume PCR buffer containing 1.5 mM MgCl_2_, 0.2 mM of each deoxynucleoside triphosphate, 1 μM each of the forward and reverse primers. An initial denaturation step of 5 min at 94 °C was conducted, followed by 35 cycles of 94 °C for 30 s, 55 °C for 45 s and 72 °C for 90 s. The procedure was completed with a final elongation step at 72 °C for 10 min. The PCR products were sequenced, and the sequences were compared with bacterial 16S rDNA sequences in GenBank using the National Center for Biotechnology Information (NCBI) Basic Local Alignment Search Tool (BLAST) program [27]. Neighbor-joining phylogenetic trees [28] were constructed using the Molecular Evolutionary Genetics Analysis (MEGA) program version 4 [29]. The reliability of phylogenetic reconstructions was estimated through bootstrap analysis (1000 replicates). The 16S rDNA sequence of the strain PA was available under GenBank accession numbers KT878384.

### 2.5. Phenol Degradation

The culture of strain PA was prepared and adjusted to an optical density at 600 nm (OD_600_) of 1.0, then the final concentration of 2% (v/v) inoculums were inoculated into the flasks containing MSM media with phenol as sole carbon source. The range of phenol concentrations was increased from 200 to 1700 mg·L^−1^. The flasks were incubated at 30 °C with 150 rpm for 2 days. Samples were collected periodically to measure the biomass and the phenol degradation. The biomass contents were monitored spectrophotometrically by measuring absorbance at 600 nm. The phenol concentrations were determined by using 4-aminoantipyrine in the colorimetric assay, according to standard methods reported by the American Public Health Association [30].

## 3. Results and Discussion

### 3.1. Isolation and Characterization of Phenol-Degrading Strains

The wastewater and sludge samples collected from an oil refinery effluent in South China were inoculated in the medium containing phenol for the enrichment and isolation of phenol-degrading bacteria. After three weeks of enrichment and one week of strain isolation, a total of 10 isolates were obtained after 24 h growth on the LB agar plates with 100 μL of a 10^5^–10^6^ fold dilution of enrichment culture. All these stains utilized phenol as the sole carbon source and energy, and 1 of the 10 isolates exhibited more growth in phenol-containing media than the others. The outstanding isolate was named as phenol-degrading strain PA and was applied in the following study. 

**Figure 1:**
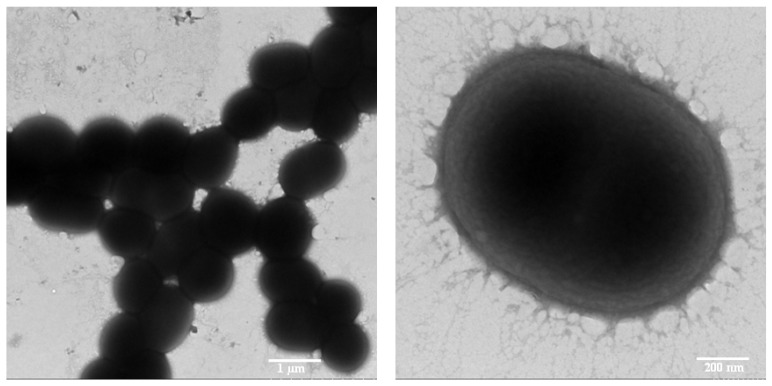
Transmission electron micrograph of *Acinetobacter calcoaceticus* PA.

The strain PA was a Gram-negative and short rod-shaped bacterium with a cell size approximately 1.2 μM in length and 0.85 μM in diameter under the microscope (Figure 1). The biochemical characteristics of strain PA were determined, and biochemical tests showed that this strain was a catalase-positive, oxidase-negative bacterium (Table 1). This strain could grow at temperatures range of 20 °C–45 °C and a wide range of pH 5–11. The optimum growth was at the condition of 30 °C and pH 8.0.

### 3.2. Identification and Phylogenetic Analysis

**Figure 2:**
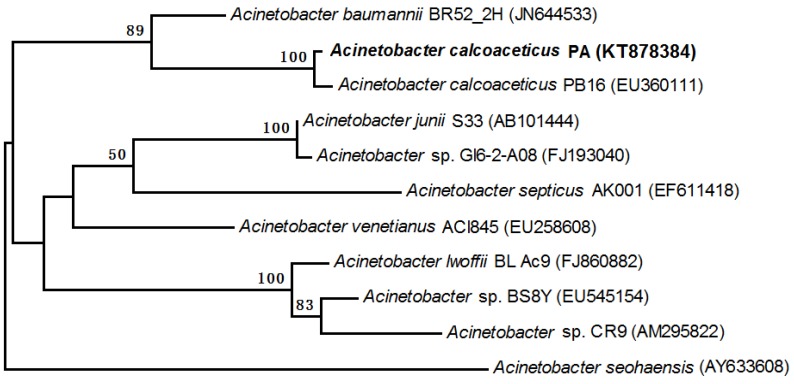
Phylogenetic relationship based on the 16S rRNA gene sequences of strain PA and related organisms from the GenBank database. Bootstrap values were calculated from 1000 replications of Kimura 2-parameter, and bootstrap values higher than 50% were shown. The scale bar represents 0.002 changes per sequence position.

The 16S rRNA gene sequences of PA were sequenced and used to construct a phylogenetic tree for further analysis. The partial sequence of 16S rRNA gene was a continuous stretch of 1424 bp. The similarities between the PA sequence and the bacterial sequences deposited in the GenBank databases were calculated, and the PA sequence showed 99% similarity to that of *A. calcoaceticus* PB16. The phylogenetic analysis (1400 unambiguous bases aligned) revealed that the strain was classified in the *Acinetobacter* genera, which belong to the *Gammaproteobacteria* sub-phylum. Based on the neighbor-joining methods, a phylogenetic tree was constructed which indicated that the closest relative of strain PA was *A. calcoaceticus* PB16 (Figure 2). Therefore, the strain PA was identified and affiliated with *A.* *calcoaceticus*.

The *Acinetobacter* species were versatile in the biodegradation of various pollutants because of its highly hydrophobic cell surface which is adhesive to solid surfaces [32]. *A. calcoaceticus* is known to be a Gram-negative aerobic bacteria utilizing phenol as the sole source of carbon and energy [8,9]. *A. calcoaceticus* PHEA-2, isolated from phenol polluted wastewater in China [33], has been used for the complete genome analysis because of its high ability to effectively degrade phenol in the bioremediation of phenol-polluted wastewater [34].

### 3.3. Phenol Biodegradation by Acinetobacter Calcoaceticus PA

**Figure 3:**
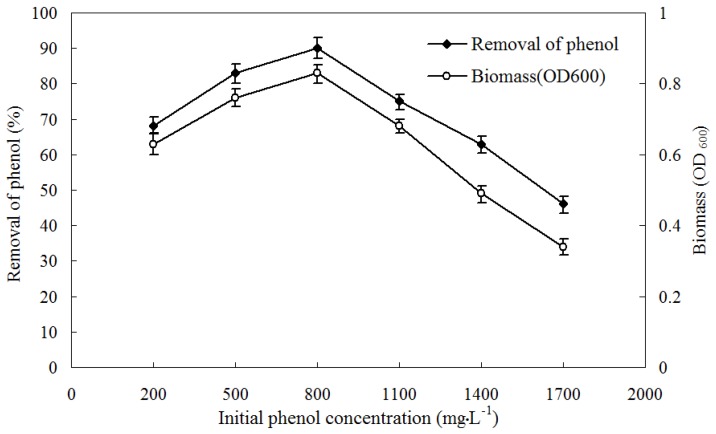
Profile of bacterial cell growth and phenol degradation at various initial concentrations of phenol.

The phenol-degradation characteristics and biomass of *A. calcoaceticus* PA at various initial concentrations of phenol (200–2500 mg·L^−1^) were determined by monitoring phenol concentration and cell growth at OD_600_ periodically. The maximum biomass and degradation of phenol were observed at the initial phenol concentration of 800 mg·L^−1^ (Figure 3). An inhibitory effect showed that the biomass growth and the degradation of phenol were declined with the elevated initial phenol concentration higher than 800 mg·L^−1^. The removal rates of phenol were above 75% at the initial phenol concentration ranging from 500 to 1100 mg·L^−1^. While there was no growth of phenol-degrading bacteria when the initial phenol concentration was higher than 2000 mg·L^−1^. The strain could grow on phenol up to a concentration of 1700 mg·L^−1^ with the degradation rate of 46.2%. 

**Figure 4:**
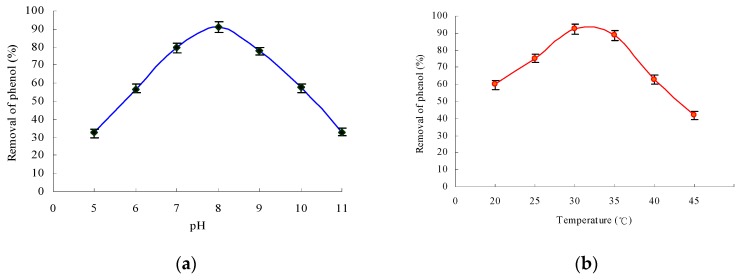
Effect of pH and temperature on phenol biodegradation (initial phenol concentration was 800 mg·L^−1^). (**a**) Effect of pH on the removal of phenol; (**b**) effect of temperature on the removal of phenol.

The effects of factors such pH values and temperature on the degradation were investigated. The bacterial strain could grow within a range of pH 5–11 (Figure 4a), and the degradation of phenol was above 78% in the range of pH 7–9. The optimum pH for phenol degradation was 8.0. These results showed that the bacterial growth of strain PA and the degradation of phenol (above 70%) were favored at temperatures of 25 °C–37.5 °C (Figure 4b). The biomass and phenol degradation reached the maximal values at a temperature of 30 °C, and both values showed only a slight change when the temperature was increased to 35 °C. On the contrary, the phenol degradation declined sharply when the temperature reached 40 °C and over. Therefore, the optimal temperature for the growth of strain PA was 30 °C. These growth conditions of *A. calcoaceticus* PA for phenol degradation were similar to those of other *Acinetobacter* reported previously [18,35,36].

Several *Acinetobacter* strains have been reported to grow by using phenol as the sole carbon and energy source. However, suspended *A. calcoaceticus* cells were partially or fully inhibited at high phenol concentrations. For example, free *Acinetobacter* cells could only degrade phenol at an initial concentration of ≤1000 mg·L^−1^ [37]. In particular, Adav and Lee noted that *A. calcoaceticus* isolated by them was fully inhibited at a phenol concentration >1500 mg·L^−1^ [38]. In our study the inhibition limit of strain *A. calcoaceticus* PA was as high as the phenol concentration of 2000 mg·L^−1^.

## 4. Conclusions

In conclusion, a bacterial strain capable of degrading phenol was isolated from an oil refinery effluent in South China, and it was identified as *Acinetobacter calcoaceticus* PA based on the 16S rDNA sequence and the phylogenetic analysis. *Acinetobacter calcoaceticus* PA has the ability to grow in a liquid medium with phenol at different concentrations as the sole carbon and energy source. The strain was able to degrade 91.6% of the initial 800 mg·L^−1^ phenol and grow at the phenol concentration of as high as 1700 mg·L^−1^. The optimal growth conditions for phenol degradation of the strain were at 30 °C and pH 8.0. Regarding that native microbial species were more adaptive than non-indigenous microorganisms in polluted environments, their predominance facilitated the bioremediation of the phenol-contaminated environments. *Acinetobacter calcoaceticus* PA isolated may be used for the bioremediation of the phenol-contaminated environments in the South China regions.

## Figures and Tables

**Table 1:** Morphological and biochemical characteristics among *Acinetobacter* strains.

Characteristics	*Acinetobacter*		
	Strain PA (This Paper)	PND-4 [31]	PND-5 [31]
Color of colonies	white	milk white	milk white
Morphology	short rod	ND	ND
Motility	−	ND	ND
Gram straining	−	−	−
Aerobic growth	+	+	+
Starch hydrolysis	−	−	−
Catalase activity	+	+	+
Gelatin hydrolysis	−	−	−
Indole production	+	−	−
Methyl red	+	−	−
Hydrogen sulfide test	+	ND	ND

ND: not determined.
